# Supplementary material for: Simulation-free estimation of an individual-based SEIR model for evaluating nonpharmaceutical interventions with an application to COVID-19 in the District of Columbia
Source: PLoS One. 2020 Nov 10;15(11):e0241949. doi: 10.1371/journal.pone.0241949 (PMC7654811; doi:10.1371/journal.pone.0241949)
Supplement: S1 File — (PDF) [file pone.0241949.s001.pdf]

Supplementary Material for *Simulation-free estimation of an individual-based SEIR model for evaluating nonpharmaceutical interventions with an application to COVID-19 in District of Columbia*

Daniel K. Sewell and Aaron Miller

1 Sensitivity to Networks

We replicated 1,000 times our estimation of the mean epicurve using our proposed approach. Each replication used a different network, where each random network was generated according to the mechanism described in the main text. Figure 1 shows the results, where the solid line is the result shown in the paper, and the shaded region corresponds to the pointwise 95% containment intervals. This provides evidence that the results do not vary substantially regardless of which network is used, so long as the underlying generating mechanism remains the same.

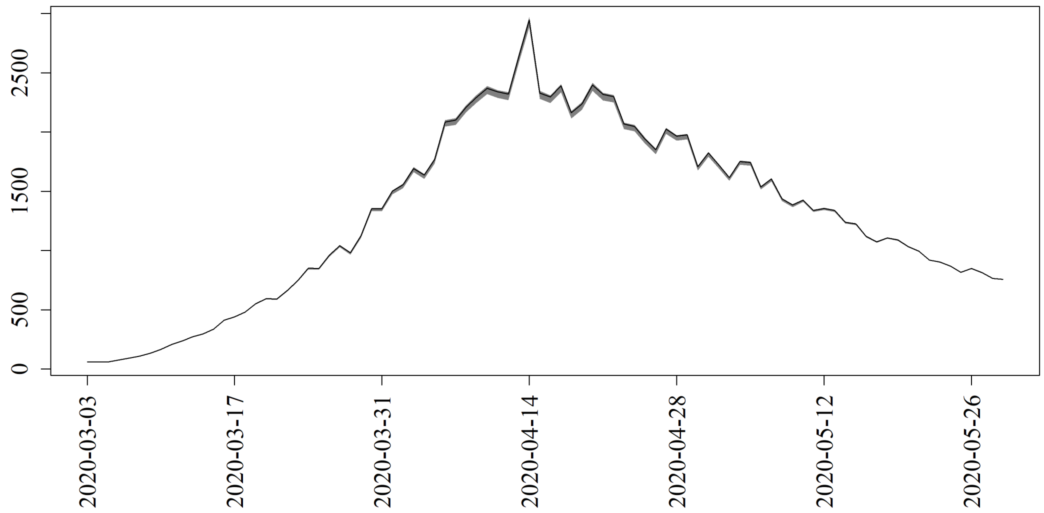

Figure 1: Sensitivity to using different networks.

2 Sensitivity to changes in parameters

We evaluated the IBM changing the parameters one at a time to determine how the expected epicurve changes accordingly. Figures 2 through 5 show the results for varying the number of days in the exposed state by ± 1, number of days in the infectious state by ± 1, the basic reproductive number by $\pm 10\%$, and the importation probability by $\pm 10\%$ respectively. Changes in these parameters lead to changes in the expected direction.

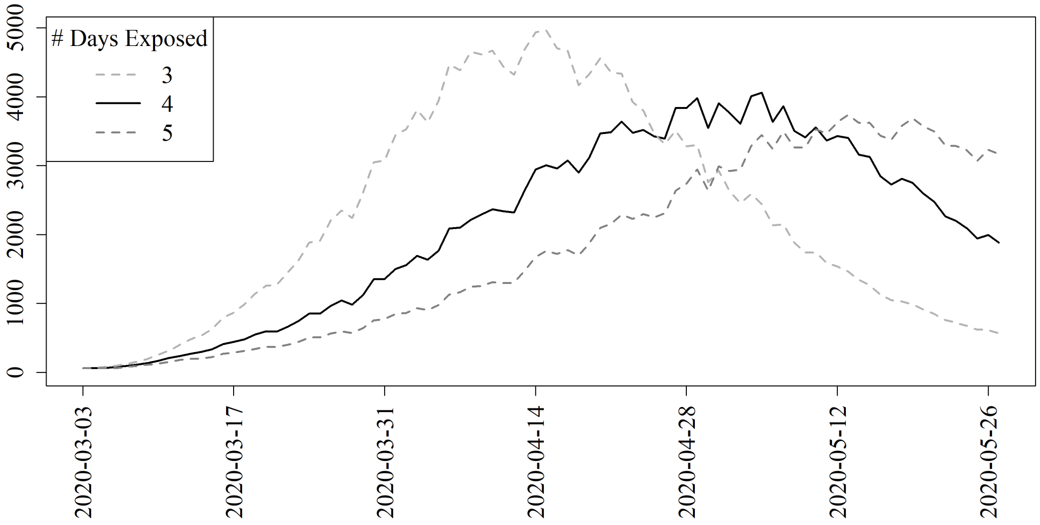

Figure 2: Sensitivity to changes in D_E

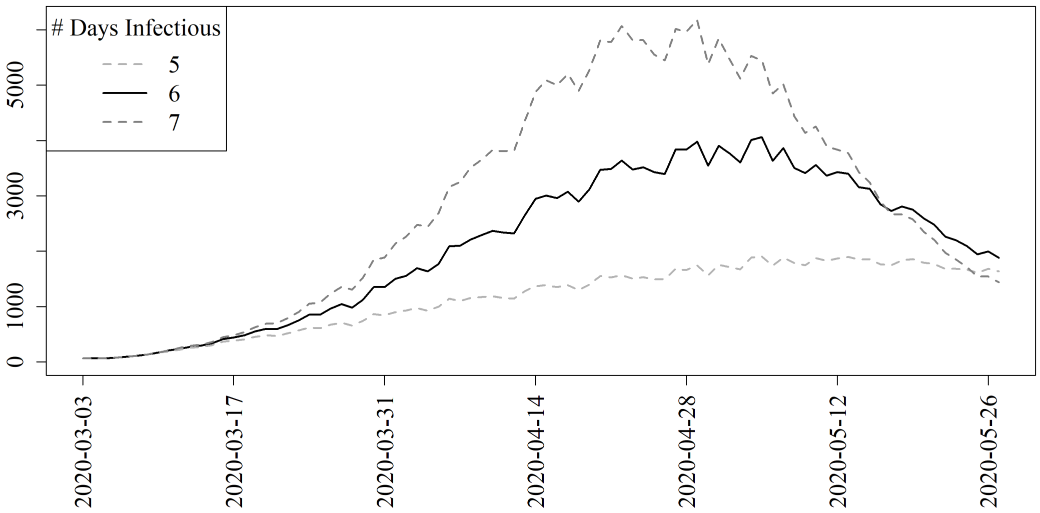

Figure 3: Sensitivity to changes in D_I

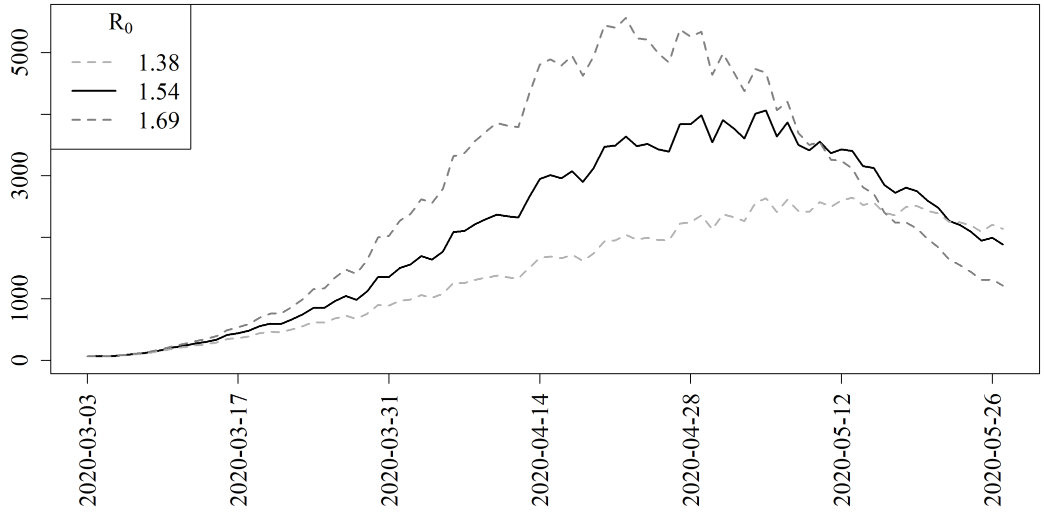

Figure 4: Sensitivity to changes in R_0

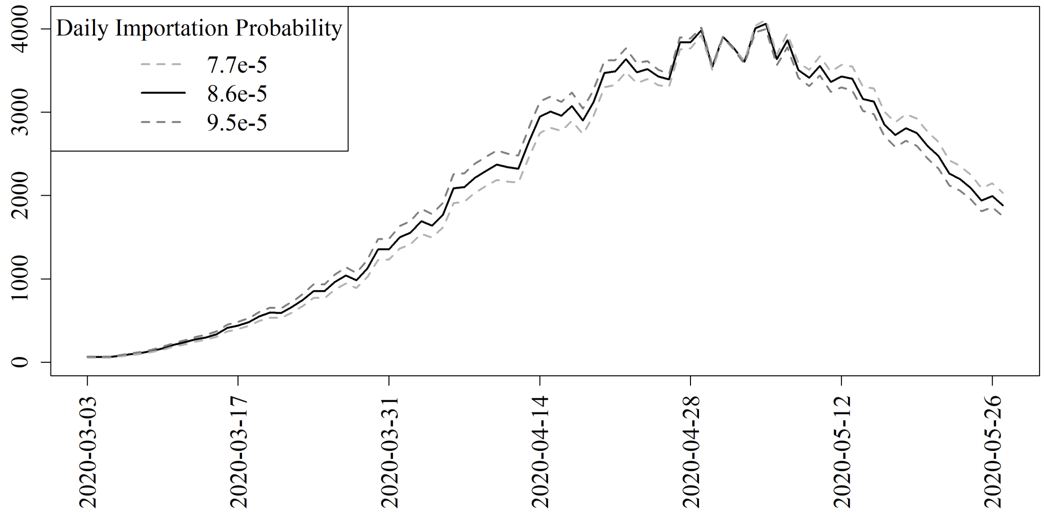

Figure 5: Sensitivity to changes in ι

3 Approximation Accuracy

We used simulation to obtain Monte Carlo estimates of the expected epicurves which we compared to those expected epicurves obtained through our proposed approach. Figure 6 shows the expected epidemic curve for Washington, D.C., as estimated from the data using our proposed approach as well as from using a Monte Carlo approximation. The data is shown alongside. It appears that during the middle of the epidemic when the infection counts are largest, there is an issue of scale, namely that our proposed approach tends to overestimate the number of infections during the peak. The shapes from the two approaches, however, remain highly similar. We also investigated our conclusions about the relative efficacy of nonpharmaceutical interventions were affected by this scaling issue. To that end, we again used Monte Carlo estimation to compare no intervention, lockdown/quarantine, face mask mandate, and both lockdown and mask mandate, where each of the interventions were implemented on March 11, 2020. Figure 7 compares our proposed approach with that of a Monte Carlo estimate. There again appears to be an issue of scale, but the overall conclusions are nearly identical.

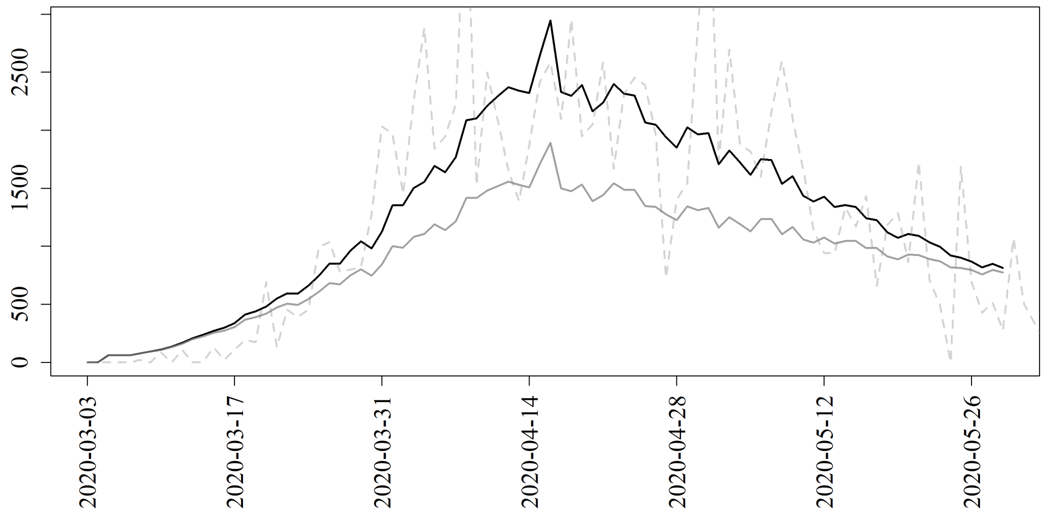

Figure 6: Accuracy of the approximation used in (5) in main text. The expected epicurve using our proposed approach is given in solid black; that from the Monte Carlo estimate is in gray; the data is given alongside in dashed gray.

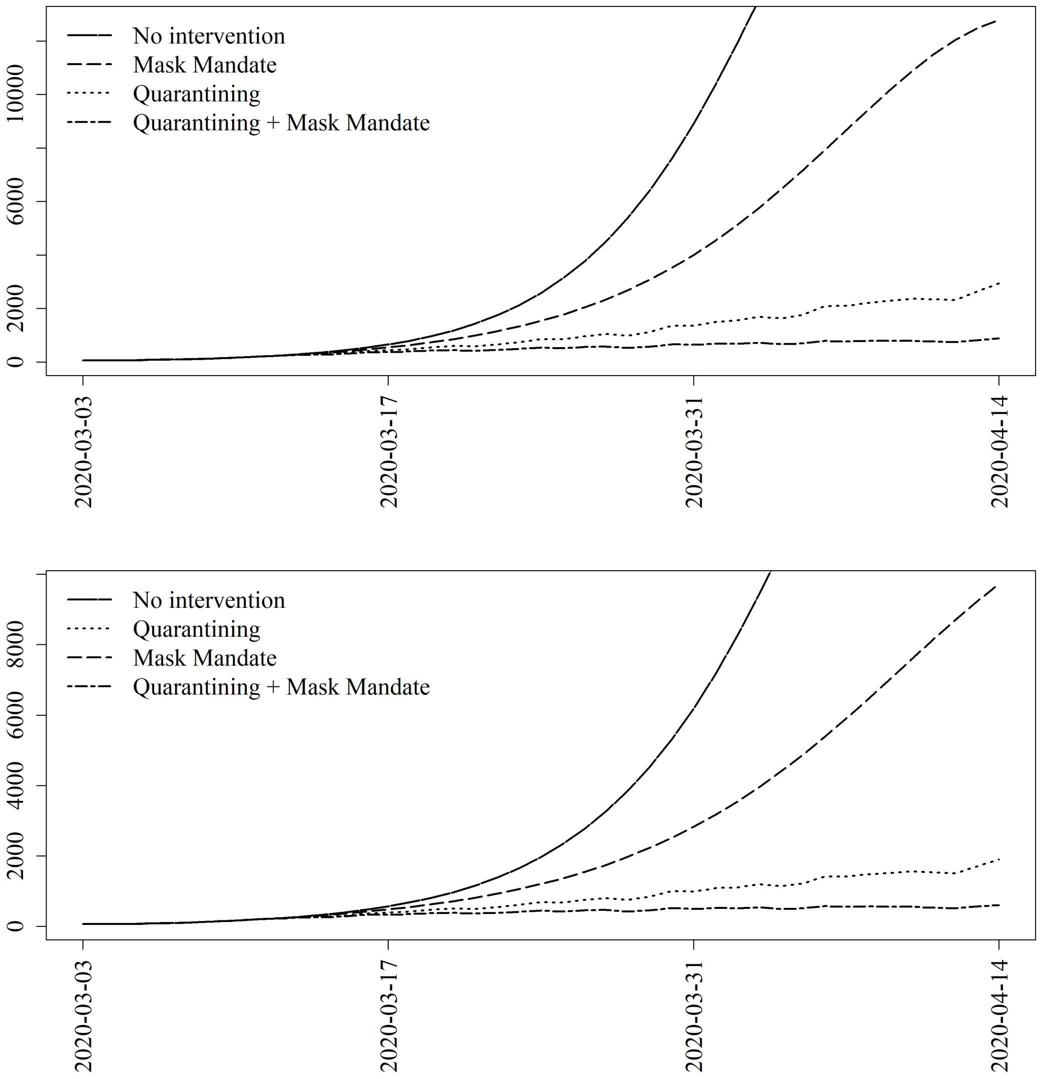

Figure 7: Accuracy of the approximation used in (5) in main text as it affects conclusions. Shown are the expected epicurves corresponding to various interventions using our proposed approach (top) and a Monte Carlo estimate (bottom).
