# Supplementary material for: Simulation-free estimation of an individual-based SEIR model for evaluating nonpharmaceutical interventions with an application to COVID-19 in the District of Columbia
Source: PLoS One. 2020 Nov 10;15(11):e0241949. doi: 10.1371/journal.pone.0241949 (PMC7654811; doi:10.1371/journal.pone.0241949)
Supplement: S2 File — (HTML) [file pone.0241949.s002.html]

marginalMeans R Package


# marginalMeans R Package

### Daniel K. Sewell

# Getting started

Download the R package at the author’s webpage and install it, or do this more directly via

```
install.packages('http://myweb.uiowa.edu/dksewell/marginalMeans_1.0.tar.gz',
                 repos=NULL,type='source')
```

Load it into R and look at the help.

```
library(marginalMeans)
```

```
help("makeNetwork") 
help("compute_SEIR")
```

In general, the most basic analysis will look something like

```
epiCurve = computeSEIR(...)
plot(epiCurve[,2],type='l') # For cumulative number of infections
plot(diff(c(0,epiCurve[,2])),type='l') # For daily number of new infections
```

# D.C. data

Let’s load in some basic libraries to help facilitate the functions’ inputs.

```
library(lubridate)
library(tidyverse)
```

We’ll look at the dates ranging from March 3 to May 27.

```
nDays = as.integer( mdy("5-27-2020") - mdy("3-2-2020") )
```

## Making a network

Users should think very carefully about what network to use. We provide functionality to obtain networks which match on the degree distribution of Kwok et al. (2018). See *Simulation-free estimation of an individual-based SEIR model for evaluating nonpharmaceutical interventions with an application to COVID-19 in the District of Columbia* for more details.

```
set.seed(123)
A = makeNetwork(N = 705749)
```

```
## 
## Attempt number 1
## 
## Attempt number 2
```

```
print(A)
```

```
## IGRAPH b1e6ad5 U--- 705684 4619602 -- Degree sequence random graph
## + attr: name (g/c), method (g/c)
## + edges from b1e6ad5:
##  [1] 1-- 15266 1-- 99484 1--148726 1--242217 1--267498 1--279281 1--309121
##  [8] 1--418969 1--452468 1--475880 1--500350 1--511647 1--579762 1--659186
## [15] 2-- 42333 2--261195 2--297764 2--369536 2--527486 2--619032 3--459387
## [22] 3--522420 3--625916 3--638820 4-- 14607 4-- 15138 4-- 35987 4-- 47123
## [29] 4-- 48106 4-- 57101 4-- 75420 4--144376 4--160882 4--167786 4--168855
## [36] 4--200909 4--200966 4--218517 4--245551 4--261626 4--301894 4--313022
## [43] 4--329153 4--329256 4--339923 4--355626 4--367632 4--367714 4--376091
## [50] 4--380476 4--381790 4--412456 4--454373 4--471566 4--475538 4--483333
## + ... omitted several edges
```

```
A_mat = as_adjacency_matrix(A)
```

## Mobility data

Get google community mobility reports via

```
mob = read_csv("https://www.gstatic.com/covid19/mobility/Global_Mobility_Report.csv?cachebust=5e35f7008c7c1554")
mob = mob %>%
  filter(country_region == "United States", sub_region_1 == "District of Columbia") %>%
  group_by(date) %>%
  summarize(retail = mean(retail_and_recreation_percent_change_from_baseline,na.rm=T)/100)
```

If the URL above fails, download global csv from https://www.google.com/covid19/mobility/.

## Get raw case counts

We can get raw case counts from The COVID Tracking Project.

```
caseData = read_csv("https://covidtracking.com/api/v1/states/daily.csv")
caseData = caseData %>% filter(state == "DC") %>%
  mutate(date = ymd(date))
caseData = caseData[nrow(caseData):1,]
caseData = caseData %>% 
  mutate(daily_cases = diff(c(0,positive)),
         daily_deaths = diff(c(0,death))) %>% 
  rename(cum_cases = positive,
         cum_deaths = death) %>%
  select(date,daily_cases,cum_cases,daily_deaths,cum_deaths)

caseData$daily_deaths[which(is.na(caseData$daily_deaths) | 
                              (caseData$daily_deaths < 0 ))] = 0
caseData$cum_deaths[which(is.na(caseData$cum_deaths))] = 0

caseData = bind_rows(tibble(date = mdy("3-3-2020") + 0:1,
                            daily_cases = 0, cum_cases = 0, daily_deaths = 0, cum_deaths = 0),
                     caseData)
```

Here’s what the data look like:

```
par(mar=c(9,6,4,1),mfrow=c(1,1))
plot(caseData$daily_cases,type='l',xaxt="n",
     xlab="",ylab="",lwd=2,cex.axis=1.5,
     main = "Number of Cases", cex.main = 1.5)
axis(1,at=seq(1,nrow(caseData),by=14),cex.axis=1.5,
     labels = caseData$date[1] - 1 + seq(1,nrow(caseData),by=14),
     las=2)
```

## Recalibrating the case counts

We can use a good estimate of the infection fatality rate (IFR) to estimate the reporting rate on a given day (again, see the accompanying Sewell & Miller manuscript for more details).

```
IFR = 0.0058
delay = 16 # Sanche et al. estimate of time from SO to death = 16.1
repRate_30day = numeric(nDays - (30 + delay - 1))
for(tt in (30 + delay):nDays){
  repRate_30day[tt - (30 + delay - 1)] = 
    ifelse(tt == (30 + delay), 
           caseData$cum_cases[tt - delay], 
           caseData$cum_cases[tt - delay] - caseData$cum_cases[tt - (30 + delay)]) / 
    ((caseData$cum_deaths[tt] - caseData$cum_deaths[tt - 30])/IFR)
}
reported_vec = c(rep(repRate_30day[1],29), 
                 repRate_30day,
                 rep(repRate_30day[NROW(repRate_30day)],delay))

caseData = caseData %>%
  filter( (date >= mdy("3-3-2020")) & (date < mdy("5-28-2020"))) %>%
  mutate(corrected_daily_cases = daily_cases / reported_vec) 

par(mar=c(9,6,4,1),mfrow=c(1,1))
plot(caseData$corrected_daily_cases,type='l',xaxt="n",
     xlab="",ylab="",lwd=2,cex.axis=1.5,
     main = "Corrected Number of Infections")
axis(1,at=seq(1,nrow(caseData),by=14),cex.axis=1.5,
     labels = caseData$date[1] - 1 + seq(1,nrow(caseData),by=14),
     las=2)
```

# Computing the expected epicurve to D.C. data

We’ll use the parameters that were estimated via OLS to fit the D.C. data.

## Quarantining

First, let’s set up the vector of time-dependent probabilities of quarantining. This is done by rescaling the google mobility by -0.7699753.

```
quarantineProb = mob %>% 
  filter(date < mdy("5-28-2020") & 
           date >= mdy("3-3-2020")) %>%
  mutate(retail = retail * (-0.7699753)) %>%
  select(retail) %>% unlist()
names(quarantineProb) = NULL
ind = which(quarantineProb > 1)
if(length(ind) > 0) quarantineProb[ind] = 1
ind = which(quarantineProb < 0)
if(length(ind) > 0) quarantineProb[ind] = 0

par(mar=c(8,5,4,1))
plot(quarantineProb,
     type='l',xaxt="n",xlab="",ylab="",cex.axis=1.5,lwd=3,
     main = "Daily probability an individual will quarantine", cex.main = 1.5)
abline(v=9,lty="13")
axis(1,at=seq(1,nrow(mob),by=7),las=2,cex.axis=1.5,
     labels=mob$date[1] - 1 + seq(1,nrow(mob),by=7))
```

## Transmission rates

Transmission rates are converted from \(R\_0 = 1.53727\) based on number of contacts per day and length of infectious period. The transmission rate is reduced by a factor of 1 - 0.7703718 to account for the mask mandate (these quantities were estimated via OLS).

```
probTransPerContact = rep(1.53727/12.5/6,nDays)
probTransPerContact[(mdy("4-15-2020") - mdy("3-2-2020")):nDays] = 
  probTransPerContact[1] *  0.7703718
```

## Running the marginal means algorithm

We can compute the expected epicurve using the compute\_SEIR() function. Change the last argument, *plotProgress* to be TRUE in order to see the daily number of infectious individuals as the algorithm is proceeding.

```
epiMean = compute_SEIR(A_mat,
                       I0 = 1,
                       nDays = nDays,
                       nDaysUntilRecovered = 6,
                       nDaysSusceptible = 3,
                       probTransPerContact = probTransPerContact,
                       importProb = 8.600991e-05,
                       propPeakToReopen = 0,
                       minNumberToReopen = NULL,
                       minDayToReopen = Inf,
                       gradualReopening = 7, #Doesn't matter unless you are doing adaptive relaxing of lockdown
                       nDaysIncreasing = Inf,
                       secondQuarProb = 0.3,#Doesn't matter unless you are doing adaptive relaxing of lockdown
                       quarantineProb = quarantineProb,
                       quarantineProb_highDegree = quarantineProb, # Doesn't matter unless you have differential quarantining based on number of contacts
                       highDeg_cutoff = Inf, # Doesn't matter unless you have differential quarantining based on number of contacts
                       plotProgress = FALSE)
```

The output is a 5 column matrix, and the rows correspond to days.

```
str(epiMean)
```

```
##  num [1:86, 1:5] 705622 705561 705500 705423 705328 ...
##  - attr(*, "dimnames")=List of 2
##   ..$ : NULL
##   ..$ : chr [1:5] "nSusc_cum" "nInf_cum" "nInf_daily" "reopening" ...
```

We can now look at the results (buffered by the 5 days we estimated it took on average for infections to be reported):

```
est_cum = c(numeric(5),epiMean[,2])
est_daily = diff(c(0,est_cum))
par(mar=c(9,6,4,1),mfrow=c(1,1))
plot(est_daily,type='l',xaxt="n",
     xlab="",ylab="",lwd=2,cex.axis=1.5,
     ylim=c(0,max(est_daily,caseData$corrected_daily_cases)),
     main = "Corrected number of infections", cex.main = 1.5)
lines(caseData$corrected_daily_cases,col=gray(0.5,0.5),lwd=2)
axis(1,at=seq(1,NROW(est_daily),by=14),cex.axis=1.5,
     labels = mdy("3-3-2020") - 1 + seq(1,NROW(est_daily),by=14),
     las=2)
```

# Looking at counterfactuals

Let’s suppose that on March 11, not only was a city-wide lockdown effected, but also a mask mandate was put into place. Further, let’s suppose that the lockdown affected only those individuals with the top 50% number of contacts.

## Transmission probabilities

Let’s create a vector reflecting a gradual rollout of a mask mandate starting on March 11.

```
startDate = as.integer(mdy("3-10-2020") - mdy("3-2-2020"))
s2 = -0.5*10^2/log(0.1)
probTransPerContact = rep(1.53727/12.5/6,nDays)
probTransPerContact[startDate:nDays] =
  probTransPerContact[1]*(
    0.7703718 + 
      (1 - 0.7703718) *
      exp(-0.5*c(0:(nDays-startDate))^2/s2)
  )

par(mar=c(9,6,4,1),mfrow=c(1,1))
plot(probTransPerContact,type='l',xaxt="n",
     xlab="",ylab="",lwd=2,cex.axis = 1.5,cex.main=1.5,
     main = "Transmission probabilities due to \n increasing use of masks")
axis(1,at=seq(1,NROW(est_daily),by=14),cex.axis=1.5,
     labels = mdy("3-3-2020") - 1 + seq(1,NROW(est_daily),by=14),
     las=2)
abline(v = startDate, col=gray(0.5,0.5))
```

## High-degree The degree distribution of the population is assumed to follow a negative binomial with mean 12.5 and dispersion parameter equal to #1.3$.

```
( highDeg = qnbinom(0.5, mu=12.5, size=1/(1 - 3/12.5)) )
```

```
## [1] 9
```

## Counterfactual epicurve

Now we can compute what we would have expected to have seen under these alternative conditions.

```
counterfactual = 
  compute_SEIR(A_mat,
               I0 = 1,
               nDays = nDays,
               nDaysUntilRecovered = 6,
               nDaysSusceptible = 3,
               probTransPerContact = probTransPerContact,
               importProb = 8.600991e-05,
               propPeakToReopen = 0,
               minNumberToReopen = NULL,
               minDayToReopen = Inf,
               gradualReopening = 7, #Doesn't matter for this context
               nDaysIncreasing = Inf,
               secondQuarProb = 0.3,#Doesn't matter for this context
               quarantineProb = rep(0,nDays),
               quarantineProb_highDegree = quarantineProb,
               highDeg_cutoff = highDeg,
               plotProgress = FALSE)

# Look at results
est_cum_cf = c(numeric(5),counterfactual[,2])
est_daily_cf = diff(c(0,est_cum_cf))
par(mar=c(9,6,1,1),mfrow=c(1,1))
plot(est_daily_cf,type='l',xaxt="n",
     xlab="",ylab="",lwd=2,cex.axis=1.5,
     ylim=c(0,max(est_daily,est_daily_cf,caseData$corrected_daily_cases)))
lines(est_daily,lwd=2,lty="13")
lines(caseData$corrected_daily_cases,col=gray(0.5,0.5),lwd=2)
axis(1,at=seq(1,NROW(est_daily),by=14),cex.axis=1.5,
     labels = mdy("3-3-2020") - 1 + seq(1,NROW(est_daily),by=14),
     las=2)
```

# Adaptive strategies

Let’s finally consider an adaptive strategy for relaxing the lockdown, and if there are too many subsequent infections, reimplementing the lockdown. Specifically, let’s relax the lockdown when the daily number of cases is less than or equal to 90% of the peak, and let’s let this relaxation rollout gradually over the course of a week (individuals probably wouldn’t start acting normally immediately just because a lockdown mandate is lifted). However, if we see 1 week of consecutive increases in the daily number of infections, we will reimplement the lockdown.

## Transmission rates

Let’s keep the transmission rates at the same observed level, where the mask mandate was put in place on April 15.

```
probTransPerContact = rep(1.53727/12.5/6,nDays)
probTransPerContact[(mdy("4-15-2020") - mdy("3-2-2020")):nDays] = 
  probTransPerContact[1] *  0.7703718
```

## Adaptive epicurve

Now we can compute what we would have expected to have seen under these adaptive conditions.

```
adaptive = compute_SEIR(A_mat,
                        I0 = 1,
                        nDays = nDays,
                        nDaysUntilRecovered = 6,
                        nDaysSusceptible = 3,
                        probTransPerContact = probTransPerContact,
                        importProb = 8.600991e-05,
                        propPeakToReopen = 0.9,
                        minNumberToReopen = NULL,
                        minDayToReopen = 45,
                        gradualReopening = 7, # How long before quarantining ceases
                        nDaysIncreasing = 7, # How many days of increasing case counts before reinstating lockdown
                        secondQuarProb = quarantineProb[NROW(quarantineProb)],
                        quarantineProb = quarantineProb,
                        quarantineProb_highDegree = quarantineProb, # Doesn't matter unless you have differential quarantining based on number of contacts
                        highDeg_cutoff = Inf, # Doesn't matter unless you have differential quarantining based on number of contacts
                        plotProgress = FALSE)

# Look at results
est_cum_adaptive = c(numeric(5),adaptive[,2])
est_daily_adaptive = diff(c(0,est_cum_adaptive))
par(mar=c(9,6,1,1),mfrow=c(1,1))
plot(est_daily_adaptive,type='n',xaxt="n",
     xlab="",ylab="",cex.axis=1.5)
polygon(x = c(5 + range(which(adaptive[,"reopening"] == 1)),
              5 + rev(range(which(adaptive[,"reopening"] == 1)))),
        y = 1e4*rep(c(-1,1),each=2),
        border = NA, col= adjustcolor("steelblue",0.5))
polygon(x = c(5 + max(which(adaptive[,"adapting"] == 0)),1e4,
              1e4,5 + max(which(adaptive[,"adapting"] == 0))),
        y = 1e4*rep(c(-1,1),each=2),
        border = NA, col= adjustcolor("tomato",0.5))
lines(est_daily_adaptive,lwd=2)
legend("topleft",col = c("steelblue","tomato"), bty = "n",cex=1.5,
       pch = 15, legend = c("Relaxing the lockdown","Reimplementing the lockdown"))
axis(1,at=seq(1,NROW(est_daily),by=14),cex.axis=1.5,
     labels = mdy("3-3-2020") - 1 + seq(1,NROW(est_daily),by=14),
     las=2)
```
